# A Reference Handbook of the Medical Sciences

**Published:** 1888-08

**Authors:** 


**A Reference Handbook of the Medical Sciences.** Embracing the entire range of Scientific and Practical Medicine and Allied Science. Volume VI., by various writers. Illustrated by chromo-lithographs and fine wood engravings. Edited by ALBERT H. BUCK, M. D. Complete in eight volumes. Price per volume, muslin, $6.00; sheep, $7.00; half morocco, $8.00. New York: Wm. Wood & Co.

The previous volumes of this valuable work have been favorably noticed in this JOURNAL. The present volume justly maintains the high character accorded to its predecessors. In alphabetical order it comprehends subjects ranging from Pra to Tep. The authors comprise the best writers on this continent, and we bespeak for their labor the earnest appreciation of the profession and the public. Such a work is above a critical review. It supplies to the practitioner of medicine the place of the encyclopœdia to the student, and gives him, in a condensed form, the main points of the subject of which it treats, by authors of eminence.

